# Large-Scale Sampling Reveals the Strain-Level Diversity of *Burkholderia* Symbionts in *Riptortus pedestris* and *R. linearis* (Hemiptera: Alydidae)

**DOI:** 10.3390/microorganisms12091885

**Published:** 2024-09-13

**Authors:** Xin-Rui Hou, Si-Ying Fu, Yuan Wang, Jia-Yue Zhou, Tian-Yi Qi, Yan-Fei Li, Wen-Jun Bu, Huai-Jun Xue

**Affiliations:** Institute of Entomology, College of Life Sciences, Nankai University, Tianjin 300071, China; 2120221473@mail.nankai.edu.cn (X.-R.H.); gefsy@nankai.edu.cn (S.-Y.F.); wyuan0526@mail.nankai.edu.cn (Y.W.); 13836198804@163.com (J.-Y.Z.); 2120231584@mail.nankai.edu.cn (T.-Y.Q.); yanfeili@nankai.edu.cn (Y.-F.L.)

**Keywords:** amplicon sequencing, co-dominance, competition, Hemiptera, microbial community, stinkbug

## Abstract

*Burkholderia* (sensu lato) is a diverse group of β-Proteobacteria that exists worldwide in various environments. The SBE clade of this group was thought to be mutualistic with stinkbugs. *Riptortus–Burkholderia* was suggested as an ideal model system for studying insect–microbe symbiosis. To explore the strain-level diversity of *Burkholderia* at the individual and population levels of *Riptortus* stinkbugs (Hemiptera: Alydidae), and to uncover the factors affecting the *Burkholderia* community, large-scale sampling of two *Riptortus* species and deep sequencing data (16S amplicon) were used in the present study. Our results showed that: (1) the proportions of facultative symbiotic bacteria *Burkholderia* were very high, with an average proportion of 87.1% in the samples; (2) only six out of 1373 *Burkholderia* amplicon sequence variants (ASVs) did not belong to the SBE clade, accounting for only 0.03% of *Burkholderia*; (3) a relatively small number of *Burkholderia* ASVs had a large number of sequences, with 22, 54, and 107 ASVs accounting for more than 1.0%, 0.1%, and 0.01% of the total *Burkholderia* sequences, respectively; (4) multiple *Burkholderia* ASVs were present in most *Riptortus* individuals, but there was one dominant or two codominant ASVs, and codominance was more likely to occur when the genetic distance between the two codominant ASVs was small; and (5) the beta diversity of *Burkholderia* was significantly different between the two host species (PerMANOVA: both Jaccard and Bray–Curtis, *p* < 0.001) and among localities (PerMANOVA: both Jaccard and Bray–Curtis, *p* < 0.001). Two-way PerMANOVA also indicated that both the host (Bray–Curtis, *p* = 0.020; Jaccard, *p* = 0.001) and geographical location (Bray–Curtis, *p* = 0.041; Jaccard, *p* = 0.045) influence *Burkholderia* communities; furthermore, Mantel tests showed that the *Burkholderia* communities were significantly correlated with the geographical distance of sample locations (R = 0.056, *p* = 0.001). Together, our findings demonstrate the fine-scale diversity of *Burkholderia* symbionts and suggest a region- and host-dependent pattern of *Burkholderia* in *Riptortus* stinkbugs.

## 1. Introduction

Insects lack metabolic pathways for the synthesis of essential amino acids and most vitamins [1,2]. Accordingly, they use two strategies to obtain missing nutrients: directly from food sources or through the synthesis of symbiotic microbial partners [1]. With piercing-sucking mouthparts, hemipteran insects (aphids, cicadas, planthoppers, leafhoppers, stinkbugs, etc.) face the challenge of a nutritionally unbalanced diet, and they cannot obtain enough nutrients (e.g., essential amino acids and B vitamins) from their food. Instead, hemipteran insects usually overcome this nutrient obstacle by harboring specific beneficial microbes in their specialized symbiotic organs, either intracellularly [3,4,5] or extracellularly [6,7,8].

*Burkholderia* sensu lato is a diverse group of Gram-negative nonfermenting β-Proteobacteria that exist worldwide in various aquatic and terrestrial environments that can be mutualistic or pathogenic to plants, fungi, and animals [7,9,10,11]. Generally, *Burkholderia* sensu lato is phylogenetically divided into the following three main clades: the pathogenic *Burkholderia cepacia* complex and *Burkholderia pseudomallei* group (BCC&P); the nonpathogenic plant-associated beneficial and environmental group (PBE); and the stinkbug-associated beneficial and environmental group (SBE) [7,12,13]. *Burkholderia* sensu lato has undergone a series of taxonomic revisions since it was proposed in 1992 [14,15,16,17,18,19]. In fact, the meaning of this group is more complex than we once thought. Under the ongoing updated classification framework, *Burkholderia* sensu lato contains three major genera, as follows: *Burkholderia* sensu stricto (i.e., the BCC&P clade); *Paraburkholderia* (i.e., the PBE clade); and *Caballeronia* (i.e., the SBE clade). In addition, there are several small genera, namely, *Robbsia*, *Mycetohabitans*, *Trinickia*, and *Pararobbsia* [20,21,22]. In the following work, *Burkholderia* refers to sensu lato unless otherwise specified.

*Riptortus–Burkholderia* was suggested as an ideal model system for studying insect–microbe symbiosis [7,12]. The SBE clade of *Burkholderia* (i.e., *Caballeronia* in some references) has been shown to be a mutually beneficial bacterium with some stinkbugs (Hemiptera: Heteroptera), mainly in Lygaeoidea and Coreoidea [7,23,24,25,26], while PBE clade (i.e., *Paraburkholderia*) has been shown in Pyrrhocoroidea [27]. *Burkholderia* consumes specific nutrients and metabolic wastes in the midgut of stinkbugs; the bacterial symbiont provides the bug with essential nutrients that are lacking in the food of the bug [28].

In these stinkbugs (however, see Itoh et al., 2014) [29], *Burkholderia* is not transmitted vertically from mother to offspring but is instead acquired from the environmental soil of each generation [30]. As with other infraorder Pentatomomorpha species, specialized “crypts” are found in the posterior midgut region of *Riptortus* stinkbugs (Coreoidea: Alydidae) that consist of a large number of sac-like structures that facilitate better colonization by symbiotic microbes [31]. The middle part of the midgut is a constricted region filled with a mucus-like matrix that has the ability to prevent nonsymbiotic or pathogenic microbes from entering the posterior midgut during the environmental acquisition of symbiotic bacteria from extremely diverse soil microbiota [13,32,33,34]. The symbiotic bacteria colonize crypts starting from a limited number of infecting bacteria acquired from the environment through feeding and a massive extracellular population is established in the midgut crypts within a few days [33,35]. Under laboratory conditions, nonsymbiotic *Burkholderia* strains and even *Pandoraea* could also stably colonize midgut crypts and benefit *Riptortus pedestris*; however, further co-inoculation experiments showed that native *Burkholderia* continuously outcompeted nonnative bacteria inside crypts, which may explain the predominance of the native *Burkholderia* symbiont in *R. pedestris* [13].

**Figure 1:**
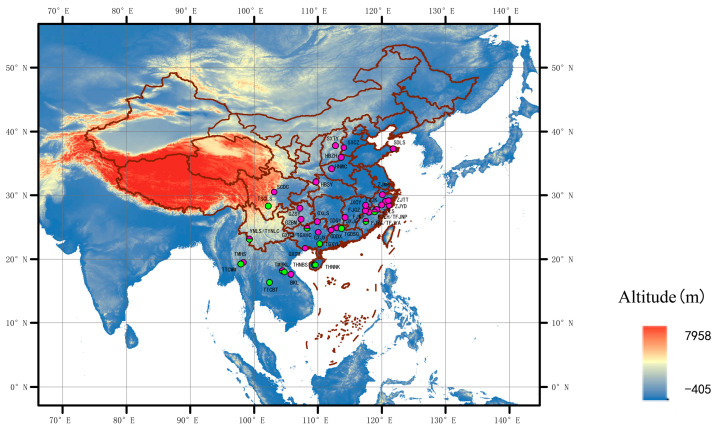
Collection sites of *Riptortus pedestris* and *R*. *linearis* (the pink dots represent *R*. *pedestris* and the green dots represent *R*. *linearis*). For details of the abbreviations in the figure, see Supplementary Table S1.

The symbiosis of *Riptortus*–*Burkholderia* symbionts at the host population level has been reported in several studies. For example, the cladistic composition of *Burkholderia* in South Korean populations of *R. pedestris* was characterized via diagnostic PCR analysis [36,37]. The species diversity of *Burkholderia* (sensu lato) in laboratory-raised *R. pedestris* individuals who provided field soil environments was analyzed by cloning sequencing [34]. Although a relaxed specificity at the strain level of *Burkholderia* in stinkbugs has been suggested [27], the symbiosis of *Riptortus–Burkholderia* has not been investigated at the bacterial strain level. Large-scale sampling of two *Riptortus* species (*R. pedestris* and *R. linearis*) (Figure 1) and deep sequencing data (16S amplicon) were used in the present study. We aim to answer the following questions: (1) What is the strain-level diversity of *Burkholderia* at the individual and population levels of the host? (2) Do host insect species and geographical distance influence *Burkholderia* communities? This study provides further insights into the symbiosis of stinkbugs and *Burkholderia* in nature.

## 2. Materials and Methods

### 2.1. Sample Collection and DNA Extraction

Adult specimens of *Riptortus* stinkbugs were collected across China, Thailand, and Laos from 2013 to 2019, preserved in 100% alcohol, and stored at −30 °C until DNA extraction. In the present study, 206 individuals of *R. pedestris* from 32 sites and 46 individuals of *R. linearis* from 13 sites (Figure 1; Supplementary Table S1) were used for DNA extraction.

The guts of alcohol-preserved specimens could not be fully detached; the whole abdominal contents were thus dissected. DNA was extracted using a Universal Genomic DNA Kit (CWBIO, Taizhou, China) according to the instructions.

### 2.2. Sequencing and Sequence Data Processing

The obtained DNA was tested for quality and then used for gene amplification. The V3-V4 hypervariable region of the 16S rRNA was amplified using the universal bacterial primer set 341f (5′-CCTAGGGRBGCASCAG-3′) and 806r (5′-GACTACNNGGGTATCTAAT-3′). The thermocycler settings were as follows: denaturation at 95 °C for 3 min; 30 cycles of denaturation at 95 °C for 30 s; primer annealing at 55 °C for 30 s; extension at 68 °C for 50 s; and a final extension at 68 °C for 10 min. The amplicons were sequenced on the Illumina NovaSeq sequencing platform at Novogene Biotech Co., Ltd, Beijing, China.

The sample data were divided according to the barcode sequence and PCR amplification primer sequence. After the amputation of barcode and primer sequences using FLASH (version 1.2.11, https://ccb.jhu.edu/software/FLASH/) [38], the reads of each sample were connected; the resulting splicing sequence was a raw tag. Fastp software (version 0.23.1) was used to strictly filter the raw tags obtained by splicing to achieve clean tags [39]. The tags acquired after the above processing were denoised by DADA2 [40] in QIIME2 (Version Qiime2-202202) [41] to yield the final amplicon sequence variants (ASVs) and feature list. Species annotation for each representative sequence of ASV was performed using QIIME2 in silva 138.1 (Silva database https://www.arb-silva.de/ for 16S) [42].

### 2.3. Bacterial Composition Calculation

The total bacterial sequences obtained from 252 *Riptortus* individuals were calculated. Most of the samples attained high sequencing data outputs. To make more efficient use of the data, the samples with sequencing depths less than 10,000 were excluded from further analysis. The absolute and relative contents of *Burkholderia* in the remaining samples were statistically analyzed.

### 2.4. Molecular Phylogenetic Analysis

Since the sequences of *Burkholderia* sensu lato were annotated as “*Burkholderia-Caballeronia-Paraburkholderia*” in the Silva 138.1 database, to further clarify the *Burkholderia* ASVs in *Riptortus* stinkbugs, maximum likelihood (ML) trees were constructed with 1393 sequences including the 1373 *Burkholderia* ASVs obtained in this study; 19 representative sequences of the SBE clade (i.e., *Caballeronia*); the BCC&P clade (i.e., *Burkholderia* sensu stricto); the PBE clade (i.e., *Paraburkholderia*); and one representative sequence of *Pandoraea* as an outgroup [30,43]. The sequences were aligned using MAFFT (version 7.520) according to the default settings [44]. ML analysis was performed using IQ-TREE (version 1.6.12) [45]. The best-fit model for amino acid sequence evolution was selected using Bayesian information criterion scores. Weights (BIC) were automatically selected using the ModelFinder module [45]. In the analysis, 1000 ultrafast bootstrap repeats were used to evaluate branch support [46,47,48]. The resulting tree was visualized and edited in FigTree (version 1.4.4) [49].

### 2.5. Strain-Level Diversity of Burkholderia

To obtain a more accurate estimate of the strain-level diversity of *Burkholderia* at the individual and population levels of the hosts, samples with fewer than 5000 *Burkholderia* reads were excluded when the data were limited to the genus *Burkholderia*.

The absolute and relative content of each *Burkholderia* ASV in the total sequences, the number of *Burkholderia* ASVs in each sample, and the relative content of each *Burkholderia* ASV in that sample, were calculated. To reflect the differences more directly, simple bar charts, pie charts, pile charts, and Venn diagrams were created. All the statistical analyses and diagrams were performed in R (version 4.3.2) using the R packages “tidyverse”, “readxl”, “scatterpie”, “ggpmisc”, and “VennDiagram”.

To estimate the genetic differentiation among major ASVs, we calculated the pairwise genetic distance of the 159 major ASVs of *Burkholderia* and calculated the genetic distance between each pair of the first and second highest ASVs in each individual using MEGAX (version 7.0.14) software [50]. To show the relationship between these major ASVs more clearly, the distribution of ASVs was summarized using a statistical parsimony haplotype network inferred in TCS 1.2.1 [51].

### 2.6. Symbiotic Microbial Community Analysis

To control for differences in sequencing depth between samples, the samples were rarefied to the minimum library size before alpha and beta diversity analyses. Flattening was performed with the R package “vegan”. The observed species richness (Sobs) index was calculated to construct rarefaction curves.

Two non-parametric richness indices, abundance-based coverage estimator (ACE) and bias-corrected Chao1, and two alpha diversity indices, Shannon (emphasizes richness and rare species) and Simpson (emphasizes evenness and dominant species) were determined for each sample. Nonparametric Mann–Whitney (for two groups) or Kruskal–Wallis (for multiple groups) tests were used to assess whether there were differences among locations and between host species for each of these estimators. SPSS (version 26.0.0) software was used for the above statistical analyses [52]. All analyses and charts were carried out in R (version 4.3.2) using the R packages “RcolorBrewer”, “dplyr”, “picante”, “vegan”, “ggprism”, “ggsignif”, “ggpubr”, and “ggplot2” [53,54].

Beta diversity comparisons were performed with the presence/absence metric Jaccard and the relative abundance metric Bray–Curtis. A PCoA map based on the Bray–Curtis index and Jaccard distance algorithm was used to show the beta diversity among the groups. ANOSIM and PerMANOVA were used to analyze the differences between host species and to compare the differences among locations (*R. pedestris*, *R. linearis*, and pooled samples were analyzed). All analyses and charts were performed in R (v4.3.2) using the packages “ggprism”, “vegan”, and “ggplot2” [53,54].

*R. pedestris* and *R. linearis* from five shared locations were selected to analyze the effects of both host species and geographic location on the structure of *Burkholderia* communities. In this analysis, two-way PerMANOVA (with 9999 permutations) was conducted using PAST software (version 4) [55], with “host species” and “location” as the main effects.

Mantel tests were used to further test whether there were correlations between *Burkholderia* communities and the geographic distance of sample sites [56,57]. *R. pedestris*, *R. linearis*, and pooled samples were analyzed. The R packages “tidyverse” and “vegan” were used to calculate the sample geographic distance and Bray–Curtis distance, respectively, and to construct a distance matrix. To directly reflect the correlation between the two factors, the R packages “ggExtra”, “ggpubr”, and “ggplot2” were applied to construct scatter plots [53,54].

Finally, the correlations between *Burkholderia* communities within the two *Riptortus* species and bioclimatic factors were tested. Nineteen bioclimatic factors with a 2.5 arc-min resolution from 1970 to 2000 on WorldClim 2.1 (https://worldclim.org/) [58] were accessed as a basic environment dataset (accessed on 3 September 2024). The correlation between each bioclimatic factor and each *Riptortus* species was tested using Mantel tests [56,57]. The analyses were performed in R (v4.3.2) using the packages “rgdal”,“raster”,“tidyverse”, and “patchwork” [53,54].

## 3. Results

### 3.1. Bacterial Composition of Riptortus Stinkbugs

**Figure 2:**
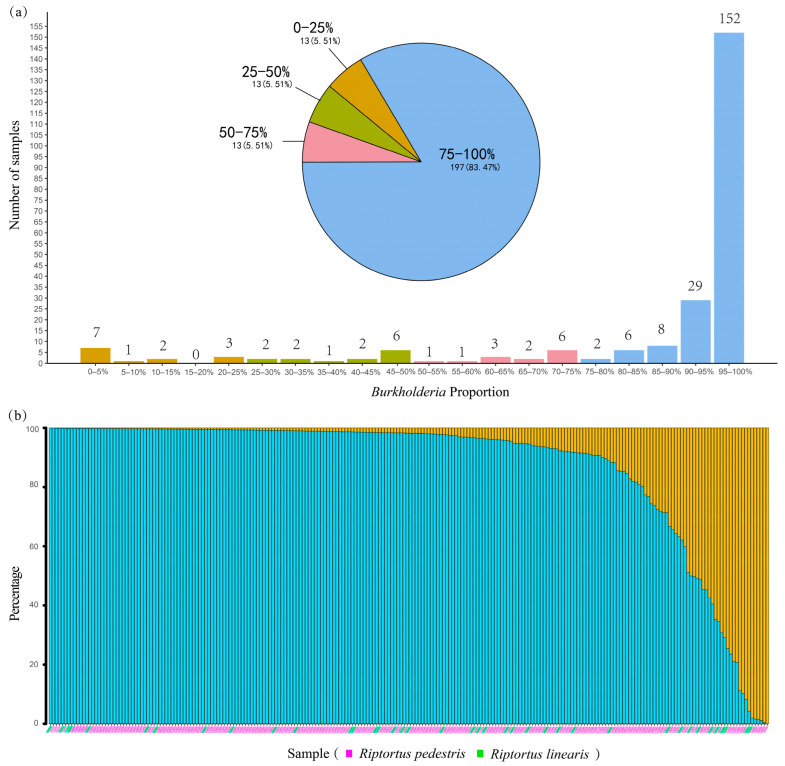
The proportion of *Burkholderia* in the total number of bacterial sequences in the *Riptortus* samples: (**a**) the bar chart and pie chart show the number of samples with different percentages of *Burkholderia* bacteria; and (**b**) bar charts show the percentage of *Burkholderia* bacteria in the total number of bacterial sequences for each sample (red represents *Burkholderia*, green represents other bacteria). The 233 samples with sequencing depths greater than 10,000 sequences are shown.

After sequence processing, quality filtering, and the removal of contaminants, a total of 12,012,828 bacterial sequences were obtained from 252 *Riptortus* individuals, with an average of 47,670 sequences (Supplementary Table S2). Most of the samples had high sequencing data outputs; 19 samples with sequencing depths less than 10,000 sequences were discarded from further analysis. In the remaining 233 samples, with an average of 51,258 sequences, a total of 12,065 bacterial ASVs were obtained. Among them, 1373 ASVs were assigned to *Burkholderia-Caballeronia-Paraburkholderia* (i.e., *Burkholderia* sensu lato). *Burkholderia* reads accounted for a high proportion of the reads in most samples, with an average proportion of 87.1%. In 181 samples (76.7%), *Burkholderia* accounted for more than 90% of the total bacterial reads. In only 26 samples (11.0%), *Burkholderia* accounted for less than 50% of the bacterial reads (Figure 2a). *Burkholderia* was undetected in only one (SXTY3) of the 233 individuals (Figure 2b). In addition to *Burkholderia*, other genera account for relatively high proportions of *Serratia*, *Enterococcus*, *Lactococcus*, *Wolbachia*, and *Bartonella* (Supplementary Table S2).

### 3.2. Phylogenetic Placement of Burkholderia Associated with Riptortus Stinkbugs

**Figure 3:**
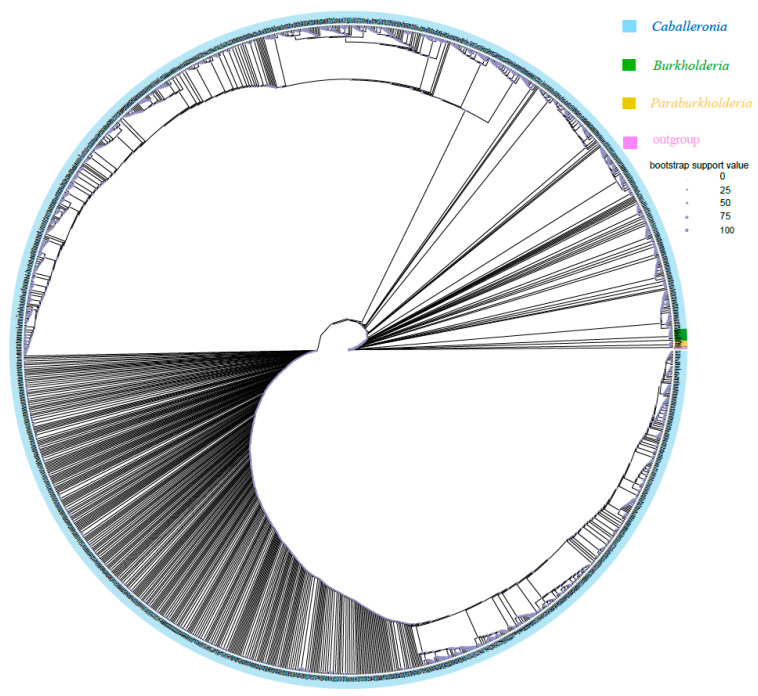
Maximum likelihood (ML) tree of *Burkholderia* reconstructed using IQ-TREE based on the V3-V4 hypervariable region of 16S rRNA (430 bp). The tree was constructed with 1393 sequences, including 1373 *Burkholderia* ASVs from *Riptortus pedestris* and *R. linearis* obtained in this study, 19 downloaded *Burkholderia* sequences from NCBI, and one representative sequence from *Pandoraea* as an outgroup. Blue, green, yellow, and pink represent the SBE clade (i.e., *Caballeronia*), BCC&P clade (i.e., *Burkholderia* sensu stricto), and PBE clade (i.e., *Paraburkholderia*) and *Pandoraea*, respectively. The size of the circle indicates the bootstrap support value.

Phylogenetic analyses based on 16S rRNA gene sequences demonstrated that most ASVs obtained in the present study (1367/1373) of *Burkholderia* sensu lato belonged to the SBE clade (i.e., *Caballeronia*) (Figure 3). Only one ASV (ASV1099, 159 sequences in total) belonged to the PBE clade (i.e., *Paraburkholderia*) and was only detected in *R. linearis*; five ASVs (ASV177, ASV337, ASV8375, ASV12112, and ASV12129; 3212 sequences in total) belonged to the BCC&P clade (i.e., *Burkholderia* sensu stricto) and were only detected in *R. pedestris.* The above two clades accounted for only 0.03% of *Burkholderia* sensu lato (11,107,919 sequences in total). However, subclades of SBE (SBE-α, SBE-β, SBE-γ, Coreoidea clade, etc.) have recently been proposed [59,60] that could not be clearly divided here, probably because the amplicon sequences were too short.

### 3.3. Strain-Level Diversity of Burkholderia

After excluding 12 samples with fewer than 5000 *Burkholderia* sequences, 11,048,782 sequences and 1373 ASVs were obtained from the remaining 221 samples (185 from *R. pedestris* and 36 from *R. linearis*). Of these 1373 ASVs, 1174 ASVs were detected in only *R. pedestris*, 81 were detected in only *R. linearis*, and 118 were shared between *R. pedestris* and *R. linearis* (Supplementary Figure S1a). Only relatively few ASVs had a large number of sequences, and the numbers of ASVs that accounted for more than 1.0%, 0.1%, and 0.01% of the total sequences were 22, 54, and 107, respectively. If an ASV exceeding 1.0% of the sequences in any host sample was retained, 160 ASVs could be obtained. Many ASVs are specific and detected in only a small number of individuals. In *R. pedestris*, 94.8% of the ASVs were present in 1–3 individuals (836, 162, and 115 ASVs were present in one, two, and three samples, respectively). Some other ASVs were detected in a greater number of individuals; for example, ASV1, ASV3, ASV16, ASV6, and ASV12 existed in more than 40 samples (Supplementary Figure S1b). Similarly, in *R. linearis*, 87.9% of the ASVs were present in one or two individuals (142 and 33 ASVs existed in one and two samples, respectively), while ASV1, ASV16, ASV19, and ASV5 were detected in more than eight samples (Supplementary Figure S1c).

To visually present the relationships between major ASVs, ASVs with a relative content of more than 1% in each sample were used to construct a haplotype network. Under this threshold, 159 ASVs were retained (141 ASVs occurred in *R. pedestris*, 64 occurred in *R. linearis*, 45 ASVs were shared, and ASV89, with only 215 bp, was excluded) (Supplementary Table S3). Among the 159 ASVs, the difference among the pairings was between 1 and 29 bases (Supplementary Figure S2; Supplementary Table S3).

**Figure 4:**
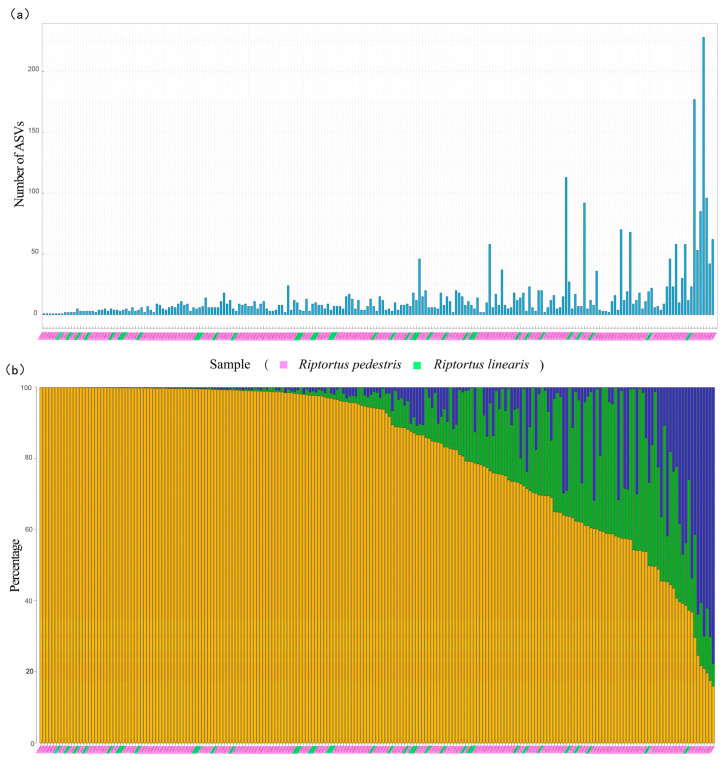
The number of *Burkholderia* ASVs and the proportions of the two most dominant *Burkholderia* ASVs in each sample of *Riptortus* species: (**a**) the number of *Burkholderia* ASVs in each sample of *Riptortus* species; and (**b**) the proportion of the two highest *Burkholderia* ASVs in each sample of *Riptortus*. The yellow bar represents the relative content of the first most abundant ASV in the sample; the green bar represents the relative content of the second most abundant ASV in the sample; and the blue bar represents the relative content of other ASVs. The pink shade on the horizontal axis represents the samples of *R. pedestris*, and the green shade represents the samples of R. linearis. The 221 samples (185 *R. pedestris* and 36 *R. linearis*) with more than 5000 *Burkholderia* reads are shown. The individuals are arranged along the horizontal axis according to the proportion of the first most abundant *Burkholderia* ASV in samples. The individuals of (**a**,**b**) (i.e., above and below) correspond one to one.

Most of the samples contained fewer than 20 *Burkholderia* ASVs (87% of the samples from *R. pedestris* and 89% of those from *R. linearis*) (Figure 4a). Although multiple *Burkholderia* ASVs were present in a *Riptortus* individual in most cases (single ASVs were detected in only six *R. pedestris* samples and one *R. linearis* sample), there were one or two dominant ASVs in most samples. In 61.6% of the *R. pedestris* individuals (114/185), and in 69.4% of the *R. linearis* individuals (25/36), the first dominant ASV accounted for more than 80% of all the *Burkholderia* sequences (Figure 4b). Overall, in 62.9% of the *Riptortus* individuals (139/221), the proportion of the first dominant ASV exceeded 80%. When the first two dominant ASVs were considered, 87.8% of these were *Riptortus* individuals (194/221) with *Burkholderia* greater than 80% (Figure 4b).

If the first two dominant ASVs occupied the majority of sequences (e.g., >80%) in the sample and were similar in number, they were called codominant ASVs. If most of the sequences in the sample belonged to one ASV, and the second ASV had significantly fewer sequences, we assumed that the first ASV had a significant advantage in terms of competition for colonization. To explore the competition pattern of symbiotic *Burkholderia* strains during the colonization process, we focused on samples containing two major ASVs to analyze the genetic differences between them (Supplementary Table S4). The samples with only one ASV or with the sum of the first two dominant ASVs comprising <80% of the entire *Burkholderia* community were treated as “no obvious codominant ASVs” and excluded from further analysis. There were 1–31 mutations between the first and second dominant ASVs in the 187 *Riptortus* individuals (Supplementary Table S4). Under different thresholds (the first dominant ASV/the second dominant ASV < 5, 3, 2, and 1.5), the proportion of codominance was significantly greater when there were fewer mutations between ASV pairs. For example, when 5 was used as the threshold, the proportion of codominance was 16.09% when the number of nucleotide mutations between ASV pairs was 11–31; the proportion of codominance was 52% when the number of mutations between ASV pairs was 1–2 (Table 1).

### 3.4. Factors Influencing Burkholderia Communities of Riptortus Stinkbugs

To ensure even sequencing depth across the samples, the dataset was subsampled to a depth of 5000 reads per sample before alpha and beta diversity analysis. All the rarefaction curves reached an asymptote, indicating adequate sequencing depth (Supplementary Figure S3).

**Figure 5:**
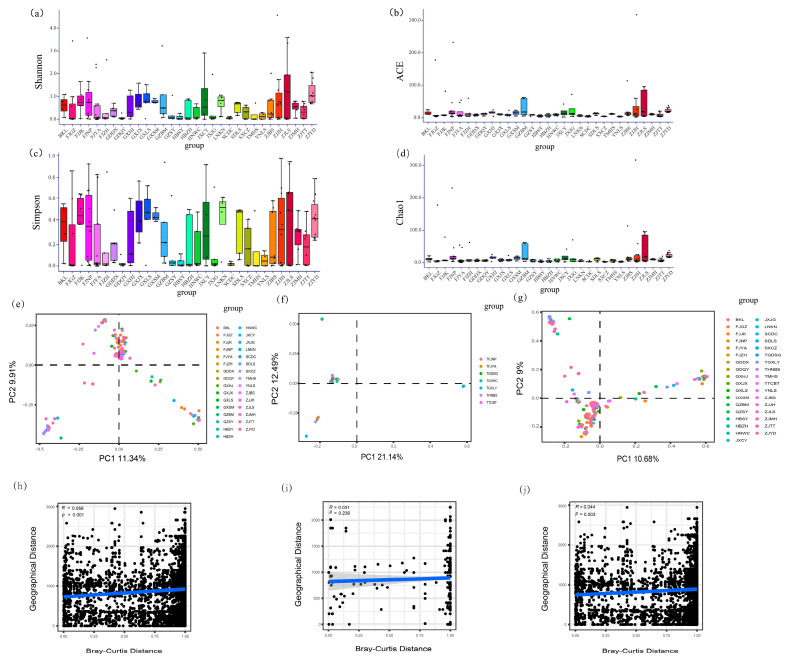
Factors influencing *Burkholderia* communities of *Riptortus* stinkbugs: (**a**–**d**) alpha diversity of symbiotic bacterial communities (including Shannon index, Chao1 index, Simpson index, and ACE index; Kruskal–Wallis tests, *p* = 0.025, *p* < 0.001, *p* = 0.089 and *p* = 0.052, respectively) in *R*. *pedestris*; (**e**–**g**) principal coordinate analysis (PCoA) of *Burkholderia* communities in *Riptortus* stinkbugs, PCoA using the Bray–Curtis distance method and based on data from *R. pedestris*, *R. linearis*, and the pooled data of both species, respectively; and (**h**–**j**) Mantel test between the *Burkholderia* community (Bray–Curtis distance) and geographic distance of the sample location using *R. pedestris*, *R. linearis*, and the pooled data of both species, respectively.

There were significant differences in two alpha diversity indicators (Kruskal–Wallis tests, Shannon, *p* = 0.025; Chao1, *p* < 0.001) but no significant differences in the other two indicators (Kruskal–Wallis tests, Simpson, *p* = 0.089; ACE, *p* = 0.052) among localities of *R. pedestris* (Figure 5a–d; Supplementary Table S5). However, none of the alpha diversity indicators differed among localities of *R. linearis* (Kruskal–Wallis tests, *p* > 0.05 in all cases) (Supplementary Figure S4; Supplementary Table S6). The results also suggested that none of the indicators differed significantly between the two host species (Mann–Whitney tests, *p* > 0.05 in all cases) (Supplementary Figure S5).

Both the ANOSIM and PerMANOVA suggested that the beta diversity of *Burkholderia* was significantly different between the two species (PerMANOVA: both Bray–Curtis and Jaccard, *p* < 0.001; ANOSIM: Bray–Curtis, *p* = 0.010, Jaccard, *p* = 0.014) (Supplementary Figure S6; Supplementary Table S7). The *Burkholderia* community in *R. pedestris* was significantly different among localities (both ANOSIM and PerMANOVA: *p* < 0.001 in all cases) (Figure 5e; Supplementary Figure S7a; Supplementary Table S8). However, there was no significant difference among localities of *R. linearis* (both ANOSIM and PerMANOVA: *p* > 0.05 in all cases) (Figure 5f; Supplementary Figure S7b; Supplementary Table S8). A significant difference among localities was suggested when the data of two species were pooled together (both ANOSIM and PerMANOVA: *p* < 0.001 in all cases) (Figure 5g; Supplementary Figure S7c; Supplementary Table S8). Further two-way PerMANOVA based on samples from two host species with the same localities indicated that localities had a significant effect on the *Burkholderia* community (Bray–Curtis, F = 1.521, *p* = 0.020; Jaccard, F = 1.311, *p* = 0.001) and that host species also had a marginally significant effect on the *Burkholderia* community (Bray–Curtis, F = 1.842, *p* = 0.041; Jaccard, F = 1.298, *p* = 0.045). However, the interaction between host species and locality had no significant effect on the *Burkholderia* community (Bray–Curtis, F = 0.854, *p* = 0.768; Jaccard, F = 0.984, *p* = 0.566) (Table 2).

Mantel tests revealed that the *Burkholderia* community of *R. pedestris* was significantly correlated with the geographical distance of the sample sites (R = 0.044, *p* = 0.003) (Figure 5h); however, this correlation was not significant for *R. linearis* (R = 0.031, *p* = 0.236) (Figure 5i). Mantel tests also showed that the *Burkholderia* community was significantly correlated with geographical distance when the data for the two *Riptortus* species were pooled together (R = 0.056, *p* = 0.001) (Figure 5j). Mantel tests also suggested that there were significant correlations between the *Burkholderia* community and several bioclimatic factors related to temperature (bio1, bio4, bio8, bio9, bio10 and bio11 for *Riptortus pedestris*; bio1, bio6, bio9 and bio11 for *R. linearis*) at the threshold of *p* = 0.01. However, there was no significant correlation between the *Burkholderia* community and any bioclimatic factor related to precipitation (Supplementary Table S9).

## 4. Discussion

We studied the diversity of *Burkholderia* in the gut of *Riptortus* stinkbugs from China, Thailand, and Laos. *Burkholderia* was detected in 232 of 233 individuals (99.6%) based on 16S rRNA gene amplicon sequencing data (Figure 2). The high infection rate of *Riptortus* was consistent with previous studies; for example, infection rates of *Burkholderia* were 97.8% in Japanese populations [61] and 80.62–93.1% in South Korean populations [36,37] of *R. pedestris* estimated by diagnostic PCR.

*Burkholderia* accounted for a very high proportion of the total bacterial sequences, with an average proportion of 87.1%. In fact, in the present study, DNA samples were extracted from the whole abdomen, which might have resulted in the detection of bacteria from abdomen regions other than the midgut M4 region; thus, the proportion of *Burkholderia* in the midgut M4 region may have been underestimated. High proportions were also found in two studies using deep sequencing methods; the genus *Caballeronia* (i.e., the SBE clade of *Burkholderia*) comprised more than 80% of the entire bacterial community in 66 field-collected individuals of *Paradieuches dissimilis* (Rhyparochromidae) [60]; and in 15 individuals of *Physopelta* species (Largidae), *Burkholderia* occupied more than 94% of the bacterial communities [27].

Phylogenetic analysis indicated that most ASVs of *Burkholderia* sensu lato belong to the SBE clade (i.e., genus *Caballeronia*); among the 1373 ASVs, only one belonged to the PBE clade (i.e., *Paraburkholderia*), and only five belonged to the BCC&P clade (i.e., *Burkholderia* sensu stricto). However, 70.4% of *R. pedestris* individuals from South Korea harbored unclassified *Burkholderia* clades, whereas 22.2% and 7.4% of individuals harbored SBE clades and BCC clades, respectively [36]. In another study based on field-collected samples from South Korea, the BCC clade was the most frequently detected clade (66.06%) among SBE, BCC and PBE; furthermore, 47.06% of *R. pedestris* individuals were found to harbor an unidentified clade [37]. The discrepancy among studies may be explained by a region-dependent pattern due to the physiological characteristics of different host populations or the differences in soil microorganisms available in different regions. Previous studies showed that different clades of *Burkholderia* provide various fitness benefits to their stinkbug hosts [13,62]. Even within the same clade (e.g., the SBE clade), not all strains are equivalently beneficial to the stinkbugs [63]. This means that the observed variation in symbiont strains could potentially result in different outcomes for stinkbug performance/fitness. In the present study, a total of 12,065 bacterial ASVs were detected in 233 samples and 1373 *Burkholderia* ASVs were detected in 221 screened samples (reads of *Burkholderia* > 5000) of *Riptortus* species. However, the number of ASVs that accounted for more than 1%, 0.1%, and 0.01% of all *Burkholderia* sequences was only 22, 54, and 107, respectively. The environment, especially the soil, contains highly diverse bacterial species, including a considerable number of *Burkholderia* species/strains, even at small spatial scales [34]. As we noted here, the DNA samples were extracted from the contents of the whole abdomen; therefore, a large variety of bacteria may be detected that enter the gut from the environment but do not colonize the M4 region of the midgut. This could explain why the diversity of the bacteria was so high while the high abundance of *Burkholderia* ASV was limited in this study. In a previous study, 341 bacterial ASVs and 61 *Burkholderia* ASVs were detected in 352 field-collected samples of *Jalysus* species [64]. The low number of ASVs in that study may be due to the lower sequencing depth, resulting in the absence of some low-abundance ASVs (6741 sequences per sample in the Ravenscraft study versus 51,258 sequences per sample in our study).

*Burkholderia* exhibit host specificity and exclusiveness when it is considered at a high taxonomic level; most *R. pedestris* individuals harbor a single clade or species of *Burkholderia* according to diagnostic PCR analysis [34,36,37]. Although competition assays have shown that native *Burkholderia* species routinely outcompete nonnative bacteria [13] and that subclade SBE-β outcompetes SBE-α [59] inside the midgut crypts of stinkbugs, multiple strains/OTUs of *Burkholderia* species are commonly detected, such as in *Physopelta* species [27], *Jalysus* species [64], and *Paradieuches dissimilis* [60]. In a recent study, bacteria belonging to one or more genera (98.65% similarity as a threshold) were also detected in individuals of *R. pedestris* by cloning methods [65]. As found in *Jalysus* [64], in our study, *Burkholderia* showed a highly promiscuous pattern in *Riptortus* stinkbugs. Using deep sequencing, more than one ASV (up to 44 ASVs) was detected in most *Riptortus* samples (214/221) (Figure 4). However, one or two dominant ASVs accounted for a high proportion of the entire *Burkholderia* community in most *Riptortus* individuals. In 52.0% of individuals, one dominant ASV accounted for more than 90% of the *Burkholderia* community, suggesting absolute dominance and an obvious competition pattern. Furthermore, in 78.3% of individuals, the first two dominant ASVs combined accounted for more than 90% of the *Burkholderia* community (Figure 4b). In the latter case, the first ASV may be clearly dominant over the second ASV in some individuals, also suggesting a competition pattern. In some other individuals, the difference in proportion between the two ASVs was not large, suggesting a codominant pattern (Supplementary Table S4). We found that the two codominant ASVs in a single host individual may be genetically far apart or differ by only one base, which to some extent supports the hypothesis that *Burkholderia* has relaxed specificity at the strain level in the stinkbug–*Burkholderia* system [27]. However, our results further suggested that the two closely related ASVs were more likely to be codominant (Table 1). This may be due to intense competition between distant strains or the host’s specific selectivity for *Burkholderia*. The relative abundances of the ASVs within individual stinkbugs could be determined by several potential drivers. As proposed above, differences in inter-ASV competitive abilities during colonization are the first one. Priority effects are another potential driver, as there is some potential for one ASV to arrive in the midgut M4 region earlier than a second ASV within a narrow time window for colonization [33,35]. The third potential driver of ASV abundances is differences in competitive ability within the midgut M4 region post-colonization. Based on the current data, it is difficult to distinguish the relative importance of these three drivers.

External factors affecting the *Burkholderia* community have been analyzed in several studies. For example, in the dock bug *Coreus margina*tus, a region-dependent pattern was suggested between European and Japanese populations [24]. Location was considered the most important factor in shaping the *Burkholderia* community in *Jalysus wickhami* and *J. spinosus* [64]. Additionally, host plant species of *Jalysus* can also lead to differences [64]. In the western conifer seed bug *Leptoglossus occidentalis,* symbiotic associations are influenced by the host rather than geography [59]. In *R. pedestris*, bacterial community structure is affected by season and geography [65]. In the present study, the PerMANOVA suggested that the beta diversity of *Burkholderia* was significantly different between the two host species and among localities. The two-way PerMANOVA also showed that both the host and geography influence the *Burkholderia* community; however, geography may play a more important role than host species (Table 2). Furthermore, Mantel tests showed that the *Burkholderia* community was significantly correlated with geographical distance (Figure 5), indicating a region-dependent pattern. It should be noted that the first two principal coordinates can explain only a small amount (approximately 20%) of the total variation, regardless of whether location or host species are considered (Figure 5e–g). A large number of individual-specific ASVs, but a small number of shared ASVs, may reduce the resolution. For *R. linearis*, the nonsignificant differences between the PerMANOVA test and the Mantel test may be due to the smaller sample size: *R. pedestris* had 183 samples, covering 31 sites, while *R. linearis* had only 28 samples, covering seven sites.

Through pairwise genetic distance calculations and haplotype network diagram analysis, we found that the dominant ASVs in samples from the same location may be the same or different, and that the base differences between unique ASVs may be large or small. Stinkbugs acquire *Burkholderia* from the environment in each generation during second instars and the microbiome varies greatly even at small spatial scales. Therefore, the association between stinkbugs and *Burkholderia* strains is very unstable [66], which might explain why samples from the same location were occupied by different strains of *Burkholderia.* Whether the colonization of symbiotic *Burkholderia* strains in the host midgut crypts is random, or if there is a density-dependent pattern of *Burkholderia* strains in competition or a host-specific selectivity for symbiotic strains, requires further study.

## Figures and Tables

**Table 1:** Codominant analysis of the first two dominant ASVs in 187 *Riptortus* individuals under multiple thresholds. The samples with only one ASV (7 samples) or with the sum of the first two dominant ASVs comprising < 80% of the entire *Burkholderia* community (27 samples) were treated as “no obvious codominant ASVs” and excluded from the analysis.

Thresholds of Codominance (the First Dominant ASV/the Second Dominant ASV)	Mutations between the First and Second Dominant ASVs	Number of Valid Samples	Number of Codominant	Codominant Percentage (%)
<5	11–31	87	14	16.09
	3–10	50	12	24.00
	1–2	50	26	52.00
<3	11–31	87	11	12.64
	3–10	50	6	12.00
	1–2	50	17	34.00
<2	11–31	87	8	9.20
	3–10	50	2	4.00
	1–2	50	13	26.00
<1.5	11–31	87	3	3.45
	3–10	50	1	2.00
	1–2	50	8	16.00

**Table 2:** The role of host species and location in structuring *Burkholderia* communities in *Riptortus* stinkbugs. The analyses were carried out using two-way PerMANOVAs and used “host species” and “locality” as the main effects.

	Bray–Curtis		Jaccard	
	*F*	*p*	*F*	*p*
Host species	1.842	0.041	1.298	0.045
Locality	1.521	0.020	1.311	0.001
Host species × Locality	0.854	0.768	0.984	0.566

## Data Availability

The original contributions presented in the study are included in the article/Supplementary Material, further inquiries can be directed to the corresponding author.

## Supplementary Materials

The following supporting information can be downloaded at: https://www.mdpi.com/article/10.3390/microorganisms12091885/s1, Figure S1: Diversity of *Burkholderia* found in *Riptortus pedestris* and *R. linearis*; Figure S2: Maximum parsimony network for V3-V4 hypervariable region of the 16S rRNA from *Burkholderia*; Figure S3: Rarefaction curves for *Burkholderia*; Figure S4: *Alpha* diversity of symbiotic bacterial communities (including Shannon index, Chao1 index, Simpson index, and ACE index) in *R. linearis*; Figure S5: Comparing of *Alpha* diversity of symbiotic bacterial communities (including Shannon index, Chao1 index, Simpson index, and ACE index) between *Riptortus pedestris* and *R. linearis*; Figure S6: Principal coordinates analysis (PCoA) of *Burkholderia* communities in *Riptortus pedestris* and *R. linearis*; Figure S7: Principal coordinates analysis (PCoA) of *Burkholderia* communities in *Riptortus* stinkbugs using the Jaccard distance method; Table S1: Sample collection information of *Riptortus pedestris* and *R. linearis*; Table S2: ASV feature table of bacteria; Table S3: Pairwise genetic distance of 159 ASVs of *Burkholderia*; Table S4: The proportion of the two highest *Burkholderia* ASVs and genetic distance between them in each sample of *Riptortus*; Table S5: *Alpha* diversity indicators of *Burkholderia* in 183 *Riptortus pedestris* samples; Table S6: *Alpha* diversity indicators of *Burkholderia* in 28 *Riptortus linearis* samples; Table S7: The role of locations in structuring *Burkholderia* communities between two *Riptortus* species; Table S8: The role of locations in structuring *Burkholderia* communities within *Riptortus* species; Table S9: The correlations between *Burkholderia* communities within two *Riptortus* species and bioclimatic factors.
