# Supplementary material for: Large-Scale Sampling Reveals the Strain-Level Diversity of Burkholderia Symbionts in Riptortus pedestris and R. linearis (Hemiptera: Alydidae)
Source: Microorganisms. 2024 Sep 13;12(9):1885. doi: 10.3390/microorganisms12091885 (PMC11434518; doi:10.3390/microorganisms12091885)
Supplement: Supplementary file 1 [file microorganisms-12-01885-s001.zip › Supplementary Figures S1-S7.pdf]

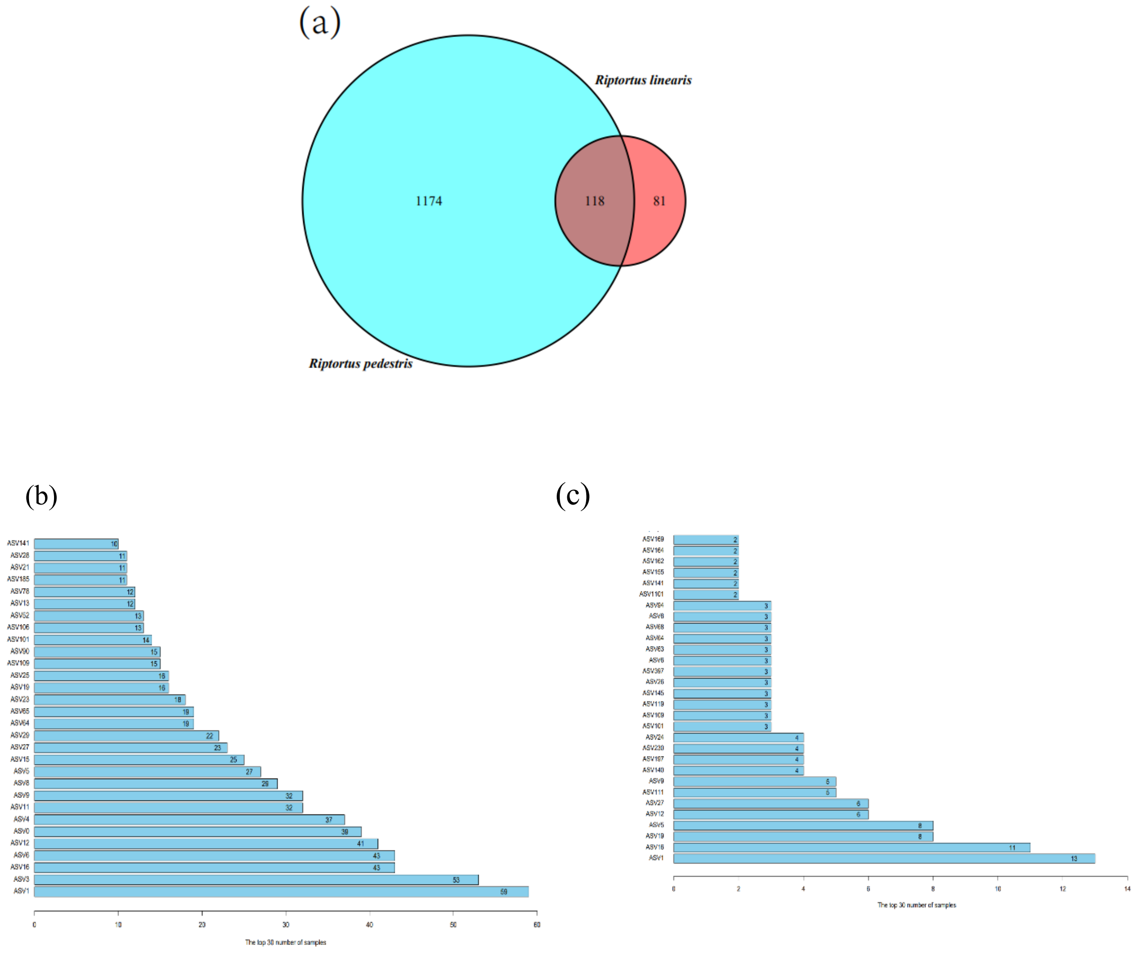

Figure S1 Diversity of *Burkholderia* found in *Riptortus pedestris* and *R. linearis.* (a) Venn diagram represents the number of shared and host species specific *Burkholderia* ASVs; (b) The number of samples of the top 30 *Burkholderia* ASVs that appear most frequently in *R. pedestris*; (c) The number of samples of the top 30 *Burkholderia* ASVs that appear most frequently in *R. linearis*.

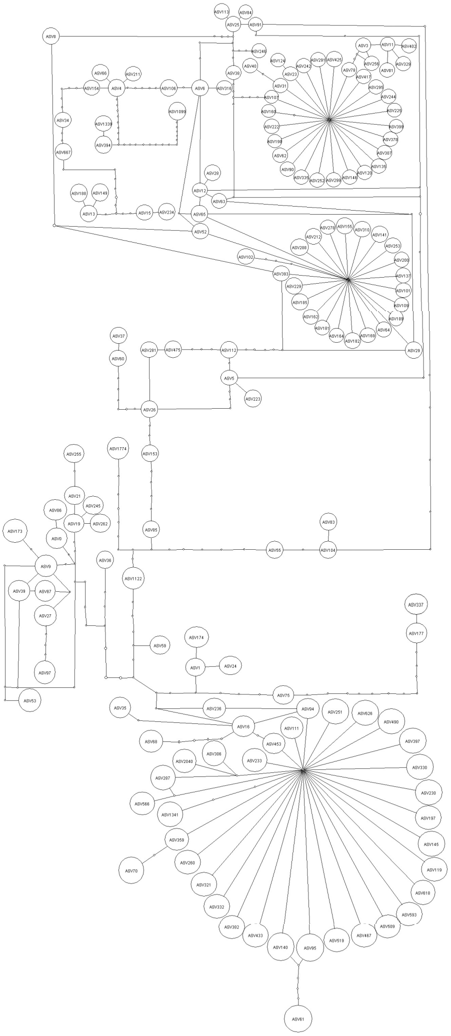

Figure S2 Maximum parsimony network for V3-V4 hypervariable region of the16S rRNA from *Burkholderia*. In total 159 major ASVs are involved. Straight lines and small dots reflect mutations and median vectors, respectively.

(a)

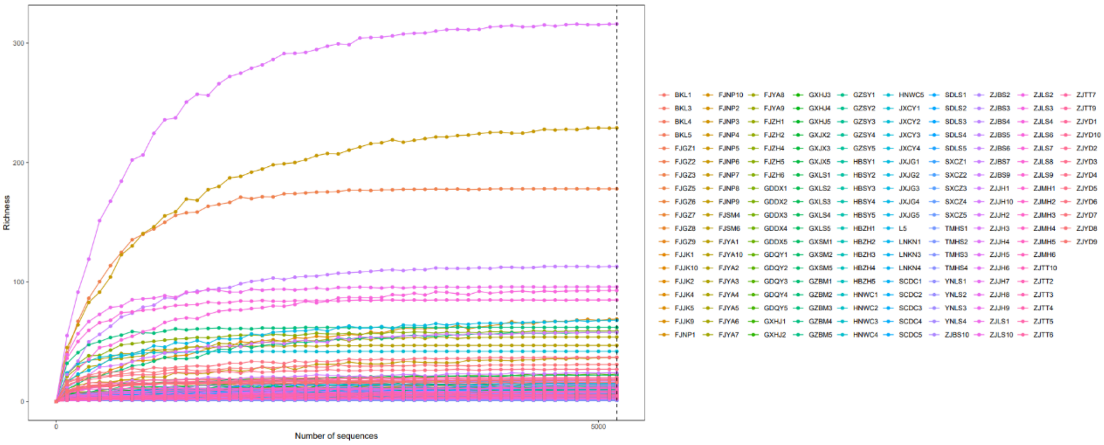

(b)

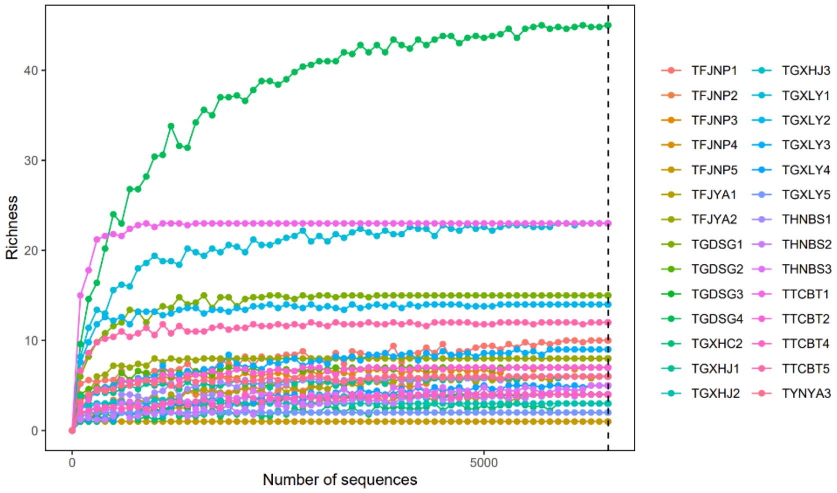

Figure S3 Rarefaction curves for *Burkholderia.* (a) samples of *Riptortus pedestris*; (b) samples of *R. linearis.*

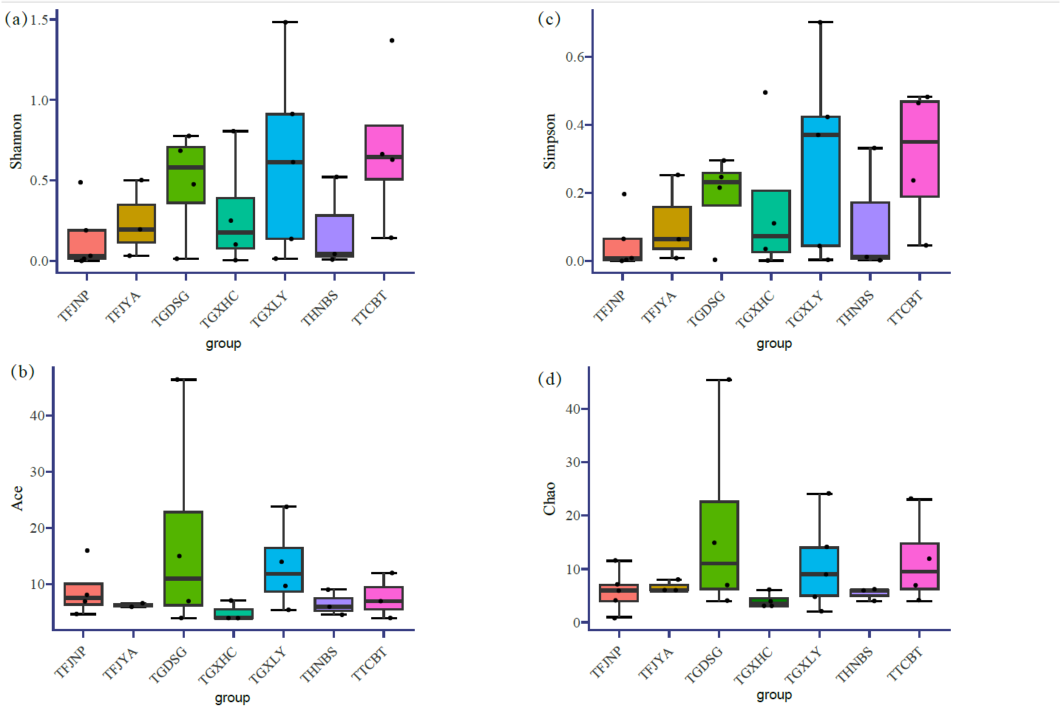

Figure S4 *alpha* diversity of symbiotic bacterial communities (including Shannon index, Chao1 index, Simpson index, and ACE index) in *R. linearis*.

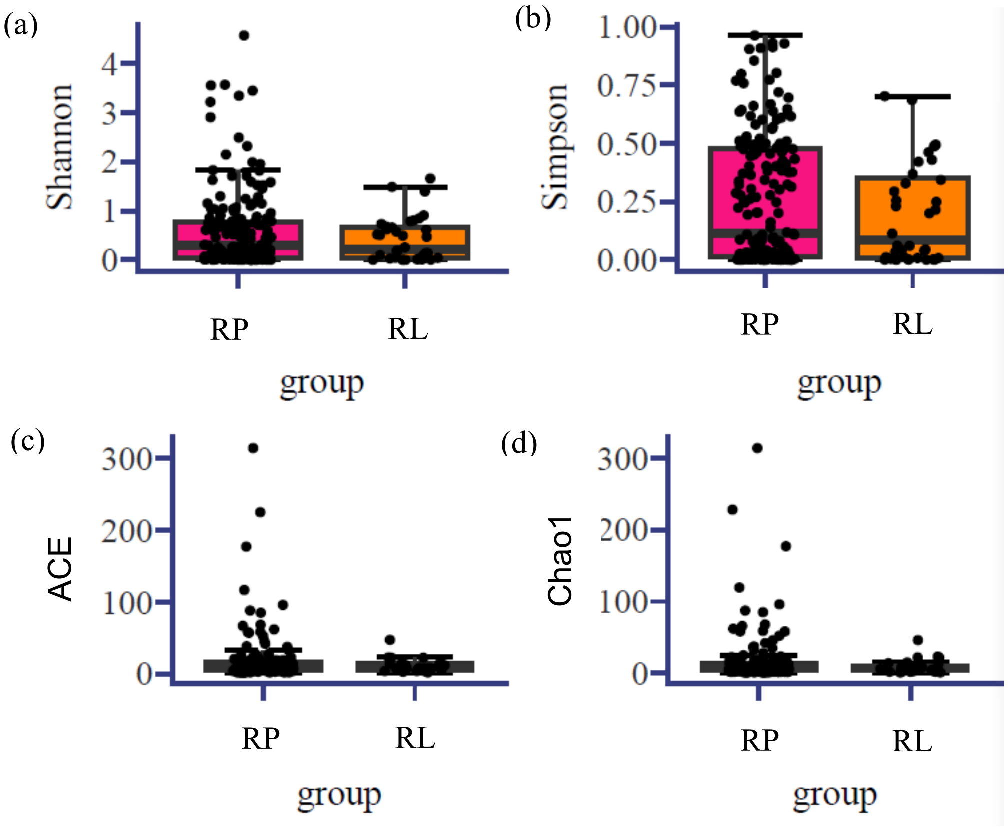

Figure S5 Comparing of *Alpha* diversity of symbiotic bacterial communities (including Shannon index, Chao1 index, Simpson index, and ACE index) between *Riptortus pedestris* and *R. linearis.* RP: *Riptortus pedestris*; RL: *R. linearis.*

(a) (b)

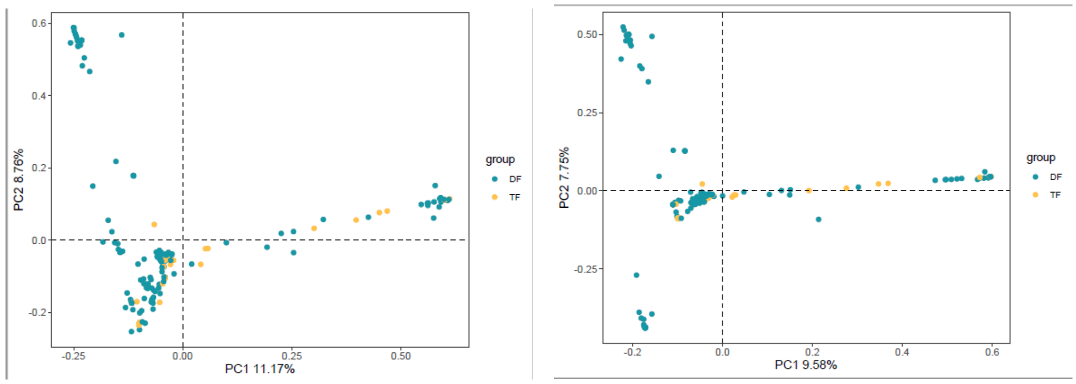

Figure S6 Principal coordinates analysis (PCoA) of *Burkholderia* communities in *Riptortus pedestris* and *R. linearis*, PCoA using the Bray-Cutis (a) and Jaccard (b) distance method, respectively.

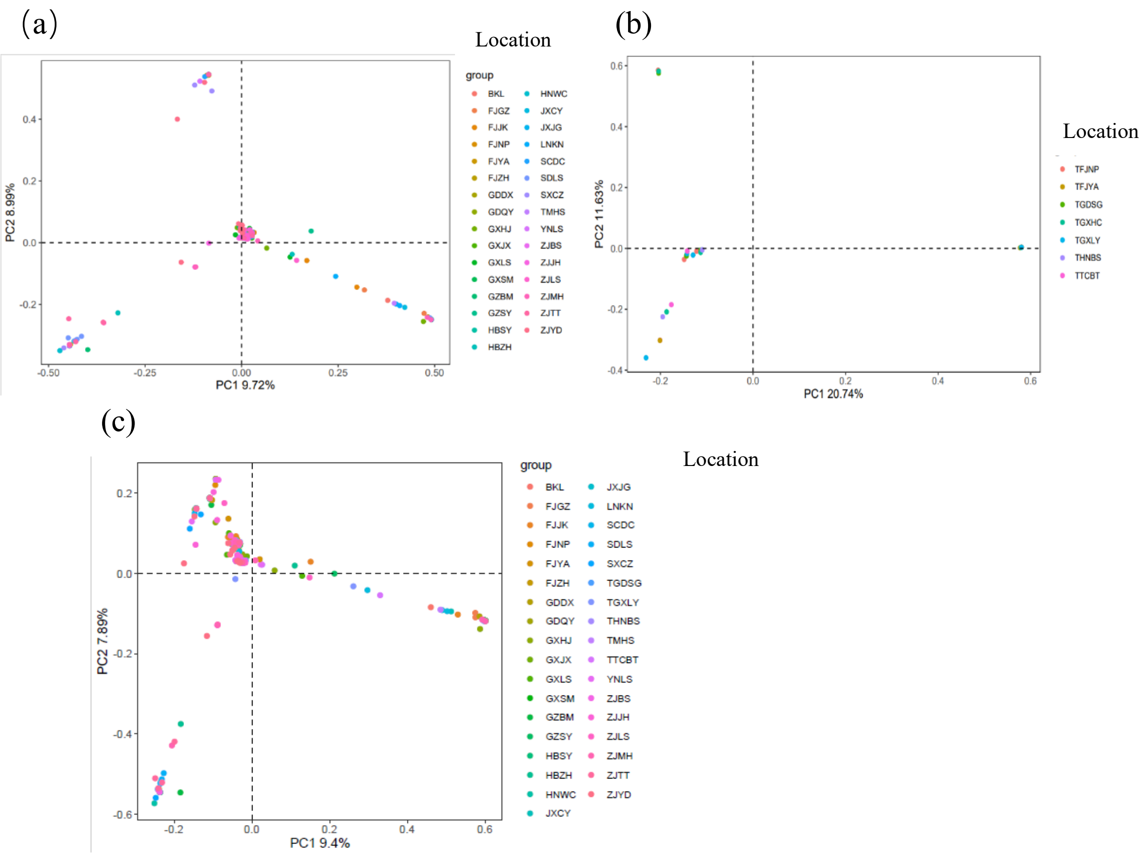

Figure S7 Principal coordinates analysis (PCoA) of *Burkholderia* communities in *Riptortus* stinkbugs, PCoA using the Jaccard distance method and based on data from *R. pedestris* (a), *R. linearis* (b) and the pooled data of both species (c), respectively.
