# Supplementary material for: Large-Scale Sampling Reveals the Strain-Level Diversity of Burkholderia Symbionts in Riptortus pedestris and R. linearis (Hemiptera: Alydidae)
Source: Microorganisms. 2024 Sep 13;12(9):1885. doi: 10.3390/microorganisms12091885 (PMC11434518; doi:10.3390/microorganisms12091885)
Supplement: Supplementary file 1 [file microorganisms-12-01885-s001.zip › Supplementary Table S5-S9.pdf]

Table S5 Alpha diversity indicators of *Burkholderia* in 183 *Riptortus pedestris* samples

Sample ID	Richness	Shannon	Simpson	Chao1	ACE
BKL1	11	0.782071	0.509843	11	11
BKL3	2	0.459683	0.285366	2	NA
BKL4	10	0.07655	0.020764	10	10.36842
BKL5	16	1.092993	0.555938	21	24.58074
FJGZ1	3	0.987119	0.602717	3	NA
FJGZ2	5	0.013507	0.003093	6	6.714286
FJGZ3	178	3.440692	0.850972	178	178
FJGZ5	6	0.565896	0.290908	6	6
FJGZ6	6	0.03576	0.00926	6	6.692776
FJGZ7	4	0.014282	0.003478	4	4.9375
FJGZ8	3	0.024319	0.006563	3	3
FJGZ9	2	0.001848	0.000387	2	NA
FJJK1	6	1.070722	0.636028	6	7.111111
FJJK10	69	1.652222	0.643536	78.71429	81.47381
FJJK2	6	0.593473	0.387015	6	6.75
FJJK4	6	0.02483	0.006179	6.5	8.782313
FJJK5	6	0.872987	0.511458	6	6
FJJK9	8	0.607467	0.370925	8	8
FJNP1	12	0.142542	0.039416	12	12
FJNP10	10	0.045791	0.011186	10.33333	11.38272
FJNP2	5	0.757038	0.511993	5	5
FJNP3	13	0.259036	0.083645	13	13
FJNP4	18	0.730059	0.311042	19	20.90384
FJNP5	3	0.012434	0.003092	3	3
FJNP6	10	1.290901	0.673215	11	14.07143
FJNP7	229	3.56698	0.912681	230.8333	232.076
FJNP8	20	1.657408	0.679403	20	20
FJNP9	37	0.860755	0.39469	46	46.28585
FJYA1	7	0.577546	0.37232	8	8.885038
FJYA10	47	2.407038	0.794285	47	47
FJYA2	7	0.062501	0.017693	7	7
FJYA3	54	2.266506	0.818559	54	54
FJYA4	6	0.667305	0.376542	6	6.515625
FJYA5	1	0	0	1	NA
FJYA6	3	0.068283	0.021876	3	NA
FJYA7	1	0	0	1	NA
FJYA8	5	0.046945	0.013098	5	5
FJYA9	4	0.194129	0.08969	4	4
FJSM6	1	0	0	1	NA
FJSM4	7	0.087245	0.025329	7	7
FJZH1	7	0.0252	0.006179	8	13.32663
FJZH2	5	0.017963	0.004251	5	5
FJZH4	59	2.614615	0.840116	62	59.89082
FJZH5	4	0.24287	0.115452	4	4
FJZH6	9	0.068545	0.017711	9	9
GDDX1	3	0.376906	0.198796	3	NA

GDDX2	5	0.707891	0.498967	5	5
GDDX3	5	0.132113	0.047846	6	NA
GDDX4	10	0.105961	0.029913	11	11.32333
GDDX5	8	0.469388	0.204464	8	8
GDQY1	6	0.034724	0.009638	9	13.6
GDQY2	4	0.060602	0.019571	4	5.09375
GDQY3	7	0.06547	0.019595	8.5	13
GDQY4	3	0.003696	0.000774	4	NA
GDQY5	6	0.272404	0.102452	6	NA
GXHJ1	23	0.118732	0.028802	25	24.40189
GXHJ2	3	0.013411	0.003478	3	4.09375
GXHJ3	12	0.290775	0.106572	13.5	14.34588
GXHJ4	14	1.022675	0.488427	14	14.43996
GXHJ5	18	1.276775	0.60489	18	18.27531
GXJX2	8	0.443041	0.20482	8	8
GXJX3	3	0.608888	0.402528	3	NA
GXJX5	12	1.588884	0.759485	12	12.29284
GXLS1	9	1.521473	0.717492	9	10
GXLS2	6	0.726569	0.477809	6	6
GXLS3	10	0.792133	0.416479	10	10.37833
GXLS4	5	0.023369	0.005794	5	5.526239
GXLS5	22	1.123082	0.596751	22.33333	22.86939
GXSM1	18	0.720195	0.432394	20.5	23.22502
GXSM2	5	0.74392	0.399143	5	5
GXSM5	15	0.909257	0.520629	15	15.30954
GZBM1	9	0.497275	0.208721	9	9
GZBM2	58	1.07631	0.390795	58.46154	59.38751
GZBM3	62	3.217789	0.928371	62	62
GZBM4	4	0.071109	0.02523	4	5
GZBM5	10	0.234029	0.084899	13	17.7037
GZSY1	8	0.025367	0.005795	8	8.5
GZSY2	7	0.023035	0.005409	10	9.944692
GZSY3	9	0.140736	0.043498	9	9
GZSY4	2	0.070888	0.026346	2	NA
GZSY5	4	1.047486	0.62877	4	4
HBSY1	2	0.003428	0.000774	2	2
HBSY2	5	0.256732	0.107741	5	6.111111
HBSY3	4	0.02913	0.007719	4	4
HBSY4	1	0	0	1	NA
HBSY5	7	0.128886	0.04593	8	8.833333
HBZH1	2	0.006319	0.001547	2	2
HBZH2	1	0	0	1	NA
HBZH3	9	0.055952	0.014639	12	12.03727
HBZH4	5	0.842743	0.504665	5	NA
HBZH5	6	0.889873	0.458994	6	NA
HNWC1	11	0.055062	0.013493	11.33333	11.86696
HNWC2	11	0.522789	0.308532	11	11.31034
HNWC3	4	0.013994	0.003478	5	8.071429
HNWC4	2	0.675354	0.482313	2	NA
HNWC5	5	0.062873	0.020702	5	5.6

JXCY1	42	2.906413	0.904914	42	42
JXCY2	11	0.834214	0.457676	11	11
JXCY3	13	0.224064	0.077163	13.25	14.30241
JXCY4	2	0.004906	0.00116	2	2
JXJG1	11	0.070373	0.018851	11.75	13.29176
JXJG2	68	1.970715	0.705661	70.54545	71.76667
JXJG3	3	0.008166	0.001933	3	4.125
JXJG4	9	0.036644	0.008877	9.2	10.75758
JXJG5	3	0.135209	0.057833	3	NA
LNKN1	3	0.953498	0.562523	3	NA
LNKN3	9	0.118015	0.034087	9	9.331815
LNKN4	4	0.698954	0.482965	4	4
L5	5	1.059872	0.614138	5	5
SCDC1	2	0.014022	0.003862	2	2
SCDC2	7	0.064407	0.01808	7	7
SCDC3	3	0.113375	0.041446	3	NA
SCDC4	3	0.018648	0.00502	3	3
SCDC5	2	0.062413	0.022572	2	NA
SDLS1	7	0.166309	0.054707	7	7
SDLS2	15	0.289774	0.112843	15	15.33902
SDLS3	12	0.740219	0.495849	13.5	13.71062
SDLS4	2	0.677147	0.484086	2	NA
SDLS5	11	0.719772	0.49976	12	12.58824
SXCZ1	3	0.324808	0.153074	3	NA
SXCZ2	2	0.519975	0.337082	2	NA
SXCZ3	1	0	0	1	NA
SXCZ4	3	0.61449	0.417856	3	3
SXCZ5	5	0.028457	0.007334	5	5
TMHS1	1	0	0	1	NA
TMHS2	7	0.015724	0.003479	10	13.24
TMHS3	3	0.003696	0.000774	4	NA
TMHS4	9	0.713863	0.493259	9	9.782313
YNLS1	1	0	0	1	NA
YNLS2	3	0.296015	0.134428	3	NA
YNLS3	2	0.011564	0.003091	2	2
YNLS4	5	0.216339	0.083252	5	5
ZJBS10	8	0.02073	0.004638	8.75	10.33333
ZJBS2	7	0.171233	0.066827	7	7.45792
ZJBS3	8	0.086465	0.02608	11	11.9375
ZJBS4	14	0.810103	0.479256	14.25	15.1981
ZJBS5	17	0.286452	0.090026	17	17.3125
ZJBS6	113	2.013561	0.582396	113.1111	113.443
ZJBS7	11	0.883647	0.518911	11	11.796
ZJBS9	4	0.007123	0.001547	4.5	7
ZJJH1	24	1.280597	0.649177	24.75	26.3931
ZJJH10	58	1.739892	0.696658	58.54545	59.71107
ZJJH2	12	0.048697	0.011572	13	13.54151
ZJJH3	4	0.008703	0.001934	4	4.75
ZJJH4	316	4.573411	0.96147	316.8077	317.3763
ZJJH5	9	0.741405	0.369981	9	9

ZJJH6	2	0.007682	0.001933	2	2
ZJJH7	5	0.59846	0.279885	5	NA
ZJJH8	3	0.088619	0.030207	3	NA
ZJJH9	5	0.775266	0.475575	5	5
ZJLS1	96	3.591926	0.933792	96	96
ZJLS10	18	1.208313	0.496855	18	18.48097
ZJLS2	3	0.006754	0.001547	3	4.111111
ZJLS3	5	0.020968	0.005023	5	5
ZJLS4	3	0.006754	0.001547	3	4.111111
ZJLS6	93	1.951429	0.647111	93.5	94.4006
ZJLS7	85	3.340174	0.91191	85	85
ZJLS8	19	1.456465	0.658245	19	19.42305
ZJLS9	6	0.058401	0.016546	6	6
ZJMH1	11	0.759968	0.308194	11	NA
ZJMH2	8	0.550543	0.317102	8	8
ZJMH3	11	0.407772	0.148763	11	11
ZJMH4	8	0.54966	0.331783	8.5	9.894444
ZJMH5	12	0.794362	0.49361	12.33333	13.24721
ZJMH6	8	0.050312	0.013104	9	9.095517
ZJTT10	3	0.204302	0.096659	3	3
ZJTT2	5	0.041784	0.011182	5	5
ZJTT3	3	0.684279	0.487276	3	3
ZJTT4	6	0.420866	0.242693	7	8.589091
ZJTT5	9	0.028027	0.006566	14	17.64583
ZJTT6	6	0.536386	0.267022	6	6
ZJTT7	4	0.018417	0.004636	4	4
ZJTT9	20	0.789722	0.310596	20	20.41356
ZJYD1	20	0.733096	0.249964	20	20
ZJYD10	16	1.152833	0.457653	16	16.60674
ZJYD2	18	1.010638	0.433432	18	18.62752
ZJYD3	18	0.676596	0.230803	18	18.61396
ZJYD4	18	0.775759	0.28145	18	18.44433
ZJYD5	27	1.591376	0.580349	27	27.34572
ZJYD6	37	1.810439	0.642086	37	37.34091
ZJYD7	18	1.032046	0.410581	18.5	20.17357
ZJYD8	31	2.060848	0.781863	31.33333	31.97404
ZJYD9	16	0.712698	0.249422	16	16

Table S6 *Alpha* diversity indicators of *Burkholderia* in 28 *Riptortus linearis* samples

Sample ID	Richness	Shannon	Simpson	Chao1	ACE
TFJNP1	10	0.487623	0.195976	11.5	16
TFJNP2	6	0.031252	0.007968	6	6.963557
TFJNP3	7	0.189495	0.064358	7	8.125
TFJNP4	4	0.012868	0.003069	4	4.703704
TFJNP5	1	0	0	1	NA
TFJYA1	6	0.031467	0.007968	6	6.613703
TFJYA2	8	0.19557	0.063215	8	NA
TYNYA3	6	0.501529	0.252253	6	6
TGDSG1	15	0.684088	0.294664	15	15
TGDSG2	7	0.475678	0.214949	7	7
TGDSG3	4	0.01307	0.003069	4	4
TGDSG4	45	0.775929	0.246199	45.375	46.37189
TGXHJ1	6	0.805585	0.4946	6	7.09375
TGXHJ2	3	0.24967	0.110178	3	NA
TGXHJ3	4	0.10165	0.03486	4	4
TGXHC2	3	0.004295	0.000921	3	4
TGXLY1	23	1.482315	0.700727	24	23.78566
TGXLY2	14	0.912094	0.369823	14	14
TGXLY3	9	0.13648	0.043869	9	9.7225
TGXLY4	5	0.013507	0.00307	5	5.444444
TGXLY5	2	0.613612	0.422596	2	NA
THNBS1	6	0.042596	0.011326	6	6
THNBS2	4	0.008296	0.001842	4	4.6
THNBS3	5	0.521198	0.33126	6	9.047619
TTCBT1	23	1.369427	0.481376	23	NA
TTCBT2	7	0.142818	0.045407	7	7
TTCBT4	4	0.66321	0.463345	4	4
TTCBT5	12	0.628412	0.236184	12	12

Table S7 The role of locations in structuring *Burkholderia* communities between two *Riptortus* species. Analyses were carried out using PerMANOVAs and ANOSIM.

Distance method	Bray-Curtis				Jaccard			
	df	R^2	F	p	df	R^2	F	p
PerMANOVA	1	0.013	2.989	< 0.001	1	0.013	2.145	< 0.001

ANOSIM	Bray-Curtis			Jaccard		
	df	R	p	df	R	p
	1	0.075	0.0096	1	0.067	0.0141

Table S8 The role of locations in structuring *Burkholderia* communities within *Riptortus* species. Analyses were carried out using PerMANOVAs and ANOSIM. "Pooled" refers to data from *R. pedestris* and *R. linearis* are pooled together.

host	Bray-Curtis				Jaccard			
	df	R^2	F	p	df	R^2	F	p
R. pedestris	30	0.241	1.577	< 0.001	30	0.223	1.468	< 0.001
R. linearis	6	0.224	0.989	0.479	6	0.221	0.938	0.769
pooled	34	0.242	1.552	< 0.001	34	0.244	1.482	< 0.001

host	Bray-Curtis			Jaccard		
	df	R	p	df	R	p
R. pedestris	30	0.167	< 0.001	30	0.181	< 0.001
R. linearis	6	-0.027	0.631	6	-0.006	0.510
pooled	34	0.153	< 0.001	34	0.156	< 0.001

Table S9 The correlations between *Burkholderia* communities within two *Riptortus* species and bioclimatic factors. A P-value in bold indicates a significant correlation at a threshold of 0.01.

		R. pedestris		***R. linearis***	
**Bioclimatic factors**		**r**	**p-value**	**r**	**p-value**
bio1	Annual Mean Temperature (℃)	0.04967	**0.001**	0.1185	0.012
bio2	Mean Diurnal Range (Mean of monthly (max temp - min temp)) (℃)	0.01765	0.132	-0.02255	0.639
bio3	Isothermality (BIO2/BIO7) (×100)	0.03019	0.025	0.06644	0.086
bio4	Temperature Seasonality (standard deviation ×100)	0.0441	**0.001**	0.1082	0.016
bio5	Max Temperature of Warmest Month (℃)	0.02762	0.014	0.09004	0.048
bio6	Min Temperature of Coldest Month (℃)	0.0353	0.015	0.1452	**0.007**
bio7	Temperature Annual Range (BIO5-BIO6) (℃)	0.02667	0.045	0.1037	0.022
bio8	Mean Temperature of Wettest Quarter (℃)	0.03916	**0.002**	0.03252	0.239
bio9	Mean Temperature of Driest Quarter (℃)	0.04277	**0.004**	0.1191	**0.008**
bio10	Mean Temperature of Warmest Quarter (℃)	0.03767	**0.003**	0.04906	0.216
bio11	Mean Temperature of Coldest Quarter (℃)	0.04283	**0.006**	0.1245	**0.006**
bio12	Annual Precipitation (mm)	0.02643	0.034	0.05449	0.194
bio13	Precipitation of Wettest Month (mm)	0.02262	0.087	0.03486	0.283
bio14	Precipitation of Driest Month (mm)	0.02262	0.078	0.0846	0.046
bio15	Precipitation Seasonality (Coefficient of Variation) (mm)	-0.0005215	0.502	0.05963	0.134
bio16	Precipitation of Wettest Quarter (mm)	0.03092	0.023	0.04249	0.25
bio17	Precipitation of Driest Quarter (mm)	0.01852	0.06	0.07539	0.047
bio18	Precipitation of Warmest Quarter (mm)	0.0235	0.082	0.02746	0.318
bio19	Precipitation of Coldest Quarter（mm）	0.02655	0.015	0.08211	0.037
